# Development and Preliminary Validation of an Equine Brief Pain Inventory for Owner Assessment of Chronic Pain Due to Osteoarthritis in Horses

**DOI:** 10.3390/ani14020181

**Published:** 2024-01-05

**Authors:** Diane L. Howard, Bryony Lancaster, Janny de Grauw

**Affiliations:** 1 Independent Researcher, 01710 Thoiry, France; dianehowardct@gmail.com; 2 The Royal (Dick) School of Veterinary Studies, University of Edinburgh, Midlothian EH25 9RG, UK; bryony.lancaster@ed.ac.uk; 3 Department of Clinical Sciences, Faculty of Veterinary Medicine, Utrecht University, 3584 CM Utrecht, The Netherlands; 4 Department of Clinical Sciences and Services, Royal Veterinary College, Hatfield AL9 7TA, UK

**Keywords:** horse, osteoarthritis, chronic pain, pain scale, questionnaire

## Abstract

**Simple Summary:**

Horses suffering from joint degeneration (osteoarthritis) often suffer pain and lameness that affect their wellbeing and welfare. Owners or caregivers observe and interact with their horses the most, but as pain and disability can vary over time, it would be valuable to have a tool to help them monitor their horses’ level of osteoarthritis pain. To this end, we designed a questionnaire. Items to be included were determined based on the scientific literature about pain expression and behaviour in horses, as well as input from discussions with owners, caregivers, and veterinary experts. We then tested the tool for ease of use and interpretation in 25 owners/caretakers of arthritic horses. Of these, 88% found the tool useful, and 84% found it easy to use. The tool took less than 5 min to complete, and readability tests showed it could be completed reliably by people with English language reading skills comparable to those with a 6th or 7th grade education. The resulting tool is undergoing further reliability testing in a larger population of owners/caretakers of arthritic and healthy horses before it can be introduced in practice.

**Abstract:**

An owner-completed questionnaire was designed to monitor the level of chronic pain and impact on quality of life in horses with osteoarthritis (OA). A standardized approach to develop and validate subjective-state scales for clinical use was followed. Scale items were generated through literature review, focus group meetings, and expert panel evaluation. The draft tool was tested for reading level and language ambiguity and piloted in 25 owners/caregivers of horses with osteoarthritis, with factor analysis performed on responses. The resulting revised questionnaire is currently undergoing validation in a larger sample population of 60 OA and 20 sound control horses. In the pilot group, 21 people (84%) found the questionnaire easy to complete and 22 people (88%) found it useful. It could be completed within 5 min by all participants. Readability scores (Flesch Reading Ease Score, Flesch–Kincaid grade level, SMOG index) indicated an English language reading level comparable to that of 6th to 7th grade in the U.S. system (age 11–12 years). Cronbach’s alpha of all items in the tool was 0.957, indicating excellent inter-item correlation. Interim analysis for 23 OA horses from the sample population showed good test–retest reliability and higher scores compared to 5 control horses. Full validation must be completed for the instrument to be used in clinical practice.

## 1. Introduction

To effectively treat pain, horse caregivers and clinicians need a way of monitoring and quantifying the amount of discomfort an animal appears to be feeling. This is particularly true of chronic pain resulting from a disease such as osteoarthritis (OA), in which the associated pain and attendant need for analgesic therapy fluctuate over time. The standard method of quantifying and monitoring pain in verbal humans is through the use of pain scales that employ numerical rating scales, visual analog scales, or verbal rating scales and are completed by the subject him or herself [1]. However, nonverbal humans, such as infants and individuals with cognitive disabilities [2,3], as well as animals, need a caretaker, preferably one who knows the patient well, to complete pain scales for them by interpreting the behavior, movement, and facial expression of the patient.

In veterinary medicine, such scales and pain monitoring instruments have been developed for several species, including laboratory animals [4], sheep [5], dairy cattle [6], and dogs and cats [7]. In dogs, instruments have been developed to monitor both acute and chronic pain [8,9], with some questionnaires, like the Canine Brief Pain Inventory (CBPI), created specifically for owner use [10]. Owner participation in monitoring chronic pain is important because the owner may be the only person who consistently sees the animal over time and is usually the person who is best able to detect subtle functional or behavioral changes in their animal [11,12]; importantly, they can alert a veterinarian when their animal needs treatment, including adjustment of the current analgesic regime.

Pain monitoring instruments currently in use for horses have primarily been designed to monitor acute pain in clinical settings [13,14,15,16,17,18,19,20,21,22,23]. A preliminary trial of a non-disease-specific Horse Chronic Pain Scale was performed recently, but the scale is yet to be validated [24]. Osteoarthritis (OA) is a major cause of chronic pain and lameness in horses [25] that is often overlooked by horse owners [26,27]. Therefore, an easy-to-complete monitoring instrument that captures the severity and quality of life impact of chronic OA pain would be very beneficial to help owners track their horse’s pain and comfort level. The goal of the present study is to create an Equine Brief Pain Inventory (EBPI) that can be easily completed by horse owners in a short period of time (5 min or less) that will be useful in monitoring both pain and quality of life, as well as treatment efficacy, and that will provide adequate information both for owners to know when to call a veterinarian and for clinicians to adjust analgesic therapy.

## 2. Materials and Methods

This project was approved by the Human Ethics Research Committee of the Royal (Dick) Veterinary School of the University of Edinburgh (HERC 379-19 and HERC 617-20) and the Morris Animal Foundation (Equine Behavior Grant ID D21EQ-508). Written informed consent was obtained from all owners or caregivers prior to participating in the study.

The steps followed in creating the EBPI are given below. These are based on published guidelines for development of clinical subjective scales [28].

### 2.1. Step 1: Item Generation

#### 2.1.1. Literature Review

A literature search was performed using PubMed, CAB abstracts, and Scopus with a date range of 2000 to 2019. The initial search terms were combinations of the words “osteoarthritis”, “horse/equine”, “pain”, “behavioral indicators”, “facial expression/scales”, “aged/geriatric”, “lameness”, “pain scales”, “owner attitudes”, and “welfare protocols”. Because of the voluminous amount of literature connected with terms such as “osteoarthritis” and “lameness” and “equine pain”, subjective filtering was performed with a bias towards leading articles with a high number of citations. PubMed “cited by” lists were also scanned manually for additional articles that might be promising for item generation. Initial chronic pain indicators meeting the criteria of not requiring physical interaction with the horse and not being specific to a given joint or type of housing were extracted. These were then used as the basis for focus group discussions.

#### 2.1.2. Focus Group Discussions

Focus group discussions were held via computer teleconference with groups consisting of both amateur and professional riders who were owners or caregivers of horses with veterinarian-diagnosed OA. Teleconferencing was used both because of the global COVID pandemic and to ensure a mix of English speakers from different geographical areas. Recruitment was through word-of-mouth and networks of the first author. All interviews began with the basic open-ended question of “What would make you think a horse had arthritis?” with responses then expanded by asking for examples and definitions. Interviews were recorded and transcribed to allow for terminology mining. A model script for focus group discussions is given in Supplementary File S1.

### 2.2. Step 2: Initial Creation of Questionnaire

A first draft of the EBPI was created based on the items generated in Step 1.

### 2.3. Step 3: Readability Evaluation

The target reading grade level for the EBPI was grade 7 or lower in the U.S. educational system. The reading level was based on U.S. National Institutes of Health recommendation that patient health information be written at a grade 6–7 reading level [29] and on the fact that the average U.S. resident reads at an 8th grade level or lower [30].

### 2.4. Step 4: Expert Evaluation and Questionnaire Revision

The first draft of the EBPI was e-mailed to nine veterinarians and/or PhDs with specialties in orthopedics, anesthesia and analgesia, equine geriatric medicine, animal behavior and zoological medicine, osteoarthritis and equine joint disease, animal welfare science, equine surgery and orthopedics, biomechanics, veterinary educational research, and clinical orthopedics and lameness for review. Expert evaluation confirmed content validity, and a revised 15-item questionnaire was created with items grouped under the headings Posture, Facial Expression, Movement, Behavior, and Based on the Above. Items were rated using an 11-point Likert scale with 0 representing normal and 10 an extreme amount of the element indicating pain or reduced function.

### 2.5. Step 5: Piloting the EBPI

Twenty-five owners or people working closely with horses with veterinarian-diagnosed OA were recruited through internet mailing lists and direct requests (convenience sample). They were asked to participate in a short teleconference session to complete the draft questionnaire, followed by a structured interview containing general questions about how their horses were kept, how the OA diagnosis was made, what treatment had been prescribed or was being used, whether they considered themselves equine amateurs or professionals, and whether they had a specific equine discipline. This was followed by questions about whether they found the questionnaire useful and easy to understand, whether any items were ambiguous, whether they would add or delete any items, and if they thought that most horse owners would be able to detect pain in their horses without first going through the posture, facial expression, movement, and behavior items.

Based on the owner interviews, the questionnaire was again revised to include a space to enter weather conditions, as well as two optional scales for additional items that the owner or veterinarian might want to monitor for a specific horse.

#### Statistical Treatment

Based on data from the pilot study, factor analysis was performed using Cronbach’s alpha calculated for questionnaire items 1–13 to check for the internal consistency of the questionnaire.

### 2.6. Step 6: Test–Retest for Scoring Reliability (Ongoing)

To confirm scoring reliability, a convenience sample of 60 owners of horses with veterinarian-diagnosed OA are being asked to complete the EBPI twice, with a two-day interval in between. Reliability between the two administrations will be assessed with the quadratic weighted k statistic and the factor analysis repeated.

### 2.7. Step 7: Validity Assessment (Ongoing)

The questionnaire will also be administered to 20 owners of horses without a history of OA or other source of chronic pain and judged to be presently sound based on video assessment to confirm that the EBPI discriminates between horses affected by chronic OA pain and sound control horses. To the extent possible, the groups will include a matched percentage of horses age ≤ 15 years and age > 15 years. Comparison will be performed using Mann–Whitney U test.

### 2.8. Step 8: Final Data Analysis and Preparation of Material for Publication

## 3. Results

### 3.1. Item Generation (Step 1)

The literature review found that the following general categories met the criteria of not requiring physical interaction with the horse and not being specific to pain originating from a given joint only: posture, position in stall, position at pasture, facial expression, and behavioral elements.

Three remote focus group sessions were held, each consisting of three participants, as well as two individual interviews. The groups included both professionals and amateurs, as well as equine bodyworkers (individuals holding some form of certification, but not necessarily qualified physiotherapists), one person specializing in equine rehabilitation, and another specializing in equine anatomy. Disciplines included leisure riding, dressage, showjumping, reining, and ground and liberty work.

Key informant interviews through informal discussions were held with 12 participants at the International Equitation Science Conference (Guelph, ON, Canada; 18–21 August 2019) and with specialists in animal behavior, including people working in zoo settings, at two conferences: The Art and Science of Animal Training Conference (Hurst, TX, USA; 22–23 February 2020) and the Progressive Equine Behavior and Training Forum (Orlando, FL, USA; 29 February to 2 March 2020). “Sampling to redundancy” [28], i.e., interviewing people until no new items were mentioned, was set as the endpoint for both the focus groups and key informant interviews, and this was achieved.

Focus group recruitment was complicated by the requirement to own or be working with a horse with veterinarian-diagnosed OA since multiple owners noted that while they were sure their horse had OA, they had not consulted with a veterinarian, while others responded that, “I take good care of my horses. None of them have ever had arthritis”.

Participants in the focus group discussions gave the following as reasons for suspecting OA and calling a veterinarian: subtle lameness that did not improve after a rest period, change in the normal gait, seeing the same tension patterns return after bodywork, changes when holding up feet for farrier, resistance to upward transitions, lameness, perceived stiffness in a gait, and not being able to do an activity (e.g., a lateral movement) that had been possible earlier.

Specific behavior changes mentioned included “grumpiness” which was clarified as “resistance to doing things that normally should be easy”, irritable facial expressions, and actions to increase distance from a person, such as bite threats, pinning ears, kicking, and bucking. Change in demeanor was also mentioned, with descriptors such as “withdrawn”, “not wanting to engage in activity they previously enjoyed”, and “not looking happy” being used.

Stances mentioned included digging hind legs into bedding, head down but the horse not eating, having a different balance on the front legs with the one leg placed outward and forward, standing with the hind legs together in a “triangular” pose, circumduction of the hind limb, and front legs out in front (haunches tucked under). Participants also noted that their horses preferred softer surfaces, found going downhill more difficult, and/or had started to short stride.

Facial expressions cited were ears to the side, eyes creased or “with a headache look”, triangular eyes, wide eyes, tight nostrils, tight lips, and tense jaw.

Other observations were horses starting to bite the walls of the stall, no longer “playing” with other horses, rubbing head and nose on the affected limb, eating less, preferring hay in a net rather than on the floor, eating at withers level and not lower, exhibiting short striding just out of the stall, and weight loss.

Behaviors that some participants never observed were the horse lying down and getting up, walking on hills, and walking on different types of ground/surface.

The key informant interviews reinforced the need to monitor behavior changes and generally referenced existing facial scales as a method of monitoring pain. Informants also brought up the need to have a baseline evaluation of an animal coupled with the danger of people setting “that’s normal for him/her” as the starting point of their assessment.

### 3.2. Item Generation (Step 2)

An initial version of the questionnaire was created based on the results of Step 1.

### 3.3. Readability Evaluation (Step 3)

For the first draft of the EBPI without the title, the Flesch Reading Ease score was 73.4 (fairly easy to read), the Flesch–Kincaid Grade Level was 5.8 (sixth grade), and the SMOG Index was 6.3 (sixth grade). These scores indicate an overall grade level of 6 (U.S. educational system), making the text suitable for individuals aged 11 years and older.

With the title, the Flesch Reading Ease score dropped to 71.6, the Flesch–Kincaid Grade Level increased to 6.1, and the SMOG Index was 6.5 (seventh grade). The overall grade level is between sixth and seventh grade, and the text should be understandable for individuals age 12 years and older.

The readability scores for the final version of the EBPI with and without the title were essentially unchanged from the first draft, with the result that the document should be accessible to horse owners age 12 years and older with a minimum of a seventh-grade education. A brief explanation of the Flesch Reading Ease and the Flesch–Kincaid Grade Level scores can be found in [31] and of the SMOG index in [32].

### 3.4. Expert Panel Evaluation (Step 4)

Supplementary File S2 summarizes changes made to the first draft of the EBPI as a result of the expert panel comments and also provides an overview of reviewer observations. A second draft of the EBPI was created based on the reviewer comments and used to pilot the questionnaire with owners and trainers of horses with veterinarian-diagnosed OA.

### 3.5. Pilot Study (Step 5)

Of the 25 participants, 16 people (64%) were amateur riders and 9 people were professional riders or trainers (36%); for the purpose of this study, professionals were defined as individuals specializing in a particular discipline who competed, judged, or taught or were actively engaged in training or rehabilitation and made a living out of these activities. The following disciplines were represented: non-competitive dressage (six people), competitive dressage (two professionals), pleasure riding (three people), various forms of groundwork (four people), Western disciplines (five professionals), cross country/eventing (two amateurs), one professional carriage driver, one professional positive reinforcement trainer, and one professional show jumping trainer. Two respondents were male.

Twenty-one people (84%) thought the questionnaire was easy to fill out, and four thought it was relatively easy to fill out. Fifteen people (60%) answered “no” to the question, “Were any of the items ambiguous or difficult to understand?” Twenty-two people (88%) said that the EBPI would be useful in monitoring their horse’s condition and two people noted that it would be useful for the “average horse owner” or “less experienced people”. One person (a non-practicing veterinarian) said that the form would not be useful for monitoring OA because it was not specific enough.

In response to the question, “Could most people identify pain without working through the various items first”, 20 people (80%) said no, while four people said yes, if the person filling out the form had a “good relationship” with the horse, and one person said definitely yes, but also observed that she lives in an area where people keep their horses at home and grow up with them.

**Figure 1:**
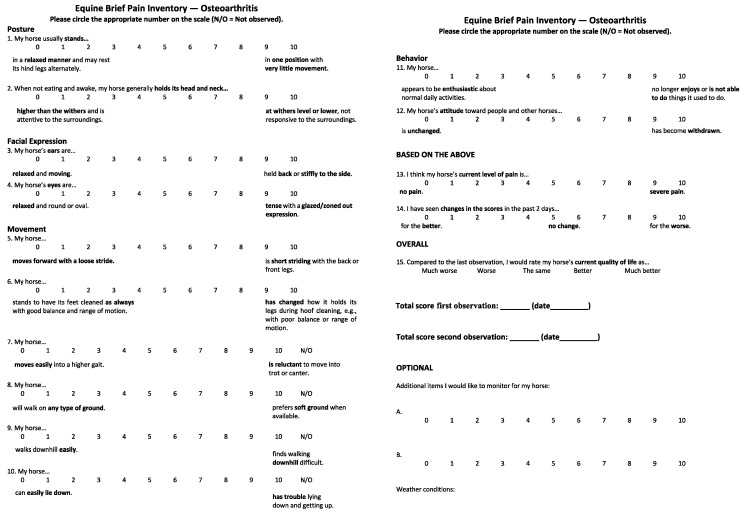
Final version of the Equine Brief Pain Inventory. Each item is scored by the owner or caregiver by selecting (circling) the score that best describes their observations for their horse for that item, with reference to the descriptors for end points of the scale (0 and 10). If owners cannot comment on a certain item for lack of observation or applicability, they can select N/O: Not Observed, for that item. The optional items include scales for postures, behaviors, or expressions that the owner finds particularly relevant for their horse, and a box to note prevailing weather conditions at the time of observation/scoring.

A revised version of the EBPI was created in response to participant comments. The major changes were adding two unlabeled optional scales for monitoring behaviors an owner felt to be important for their particular horse, and a place to enter weather conditions. The final version of the EBPI is shown in Figure 1.

Because the purpose of the questionnaire interviews was testing readability, usefulness, and ease of use, and because the interviews were conducted via teleconference without a horse present due to COVID restrictions, some participants preferred to discuss their reactions to the items and to give scores for remembered situations. As a result, the number of available data sets for analysis that were completed for a specific observed horse was limited. Cronbach’s alpha calculated for the seven responses to questionnaire items 1–13 based on horse-side collected data was 0.957.

### 3.6. Interim Analysis

At present, data have been obtained from 23 diagnosed OA horses (age 12 to 27 years), but only 19 owners (several owners had multiple horses). The owners were both amateur and professional riders, with the main disciplines being dressage and show jumping.

Scores were consistent over the two-day period in terms of range and median values (Table 1). There was some variation in individual item scores over the two-day interval, but scores divulged noticeably for a single item only, and the difference could be attributed to changes in management or weather that were mentioned during the owner interviews, suggesting that the instrument is sensitive to variations in the horse’s environment.

After the second completion of the questionnaire, the owner or caregiver participated in a brief interview about their discipline, amateur or professional status, management of the horse, how the OA diagnosis was made, and the treatment, if any, being implemented.

Except for one horse, the total scores for the five verified sound horses (age 4–16 years) were 5 points or below and were either the same or varied by only 1 point over the 2 days (see Table 2).

## 4. Discussion

The development of the pain instrument of the current report is based on the stepwise process used to validate the Canine Brief Pain Inventory (CBPI) [10] and that has been widely used for validation of health care scales [28]. Osteoarthritis was chosen as the focal disease because it is a major cause of lameness and ongoing pain [25,27] that is often overlooked by horse owners [26,27]. Providing horse owners and caregivers with a means of recognizing signs of chronic OA pain in their animals is important because for a horse to receive analgesic treatment, the owner must first realize that their animal is in pain and that the situation requires calling a veterinarian. In addition, once an analgesic regime has been established, the attending veterinarian ideally should receive data from ongoing monitoring to determine treatment efficacy and to adjust treatment according to need. Therefore, the main aim of this study was to develop an instrument that could be easily completed by horse owners and that would provide useful information to guide treatment decisions.

The original goal, to create an EBPI that would mirror the CBPI (which, in turn, is based on the human Brief Pain Inventory (BPI)), proved to be difficult for three main reasons. First, as prey animals, horses hide signs of discomfort to avoid attracting the attention of predators and herd mates. Second, many horse owners may spend only a limited amount of time per day or even per week with their animals, which reduces opportunities for observation. This is in contrast with dog owners, who often live with their dogs and so will be aware when their companion starts to have difficulty with certain activities (e.g., getting on the couch or jumping into the car). Third, many owners appear to have limited ability to read equine body language and recognize signs of pain or lameness [26,27,33,34,35]. Additional complicating factors include the course of OA, which can silently progress without signs of pain or lameness, the intermittent nature of the disease, the loose connection between disease severity and lameness [36], and the idea that OA is inevitable and almost “normal” in older horses, coupled with the notion that there are limited treatment options. However, we have tried to rigorously follow the steps used in validating the human BPI because this instrument has been recognized for decades as a powerful tool for clinical pain assessment and for the study of effective pain treatment and has demonstrated reliability and validity across languages, cultures, and disease types [37]. It is possible that the EBPI could be used with other disease conditions causing chronic pain; however, that would require separate validation for each specific disease.

After listening to the focus group discussions, the most advantageous way of structuring the EBPI to help owners recognize signs of chronic musculoskeletal pain and monitor reduced function appeared to be by starting with the posture, facial expression, movement, and behavior items to help people think about whether their horse might be in pain and how reduction in function might be affecting quality of life. The nearly unanimous agreement among the interview participants that they—or at least “ordinary horse owners”—would have difficulty in rating pain without first working through the pain and function items supports this decision.

While all of the participants in the pilot study found the questionnaire either easy or relatively easy to complete, 10 people did identify specific items that they found ambiguous. Three of these responses were about the use of the 0–10 point Likert scale, which was seen as having “too many choices”. Other comments tended to center on specific horses, breeds, or disciplines. For example, one participant noted that Item 2 (head position) could be ambiguous because Highland ponies have naturally low head carriage, while a professional carriage driver said that going downhill (Item 9) is more difficult for all horses pulling carriages. One person thought that Item 5 (short striding) needed “to be more specific” because her horse was only stiff in the morning. Other people mentioned not being sure about the time frame for observation, which we have addressed in the current instructions. Yet, another participant anticipated the finding that owner interaction can interfere with reading the horse’s expression, saying that, “because he lives out and I go to see him, facial expression can be difficult to interpret because of treats, food, and attention”. Several people noted that having photographs for facial expressions and posture items would be helpful, and we plan to incorporate images in the final version of the instrument.

The additional items suggested by participants during the pilot study confirmed that sampling to redundancy was reached during the focus group and key informant interviews. Because “weather conditions” was the addition most frequently mentioned (seven people [28%]) and because some relationship between weather and chronic pain has been demonstrated in humans (e.g., [38]), a space for noting the temperature and weather conditions has been added to the final version of the EBPI. Adding two unlabeled optional scales to the questionnaire allows people to monitor behaviors specific to their horse’s individual situation (e.g., tripping and toe dragging), preferably in consultation with their veterinarian. This same notion of making use of individual motivation to perform certain activities or tendency to display certain behaviors underlies the Client Specific Outcome Measures (CSOM) chronic pain scales for dogs and cats [39,40], where the ability and willingness to display a set of behaviors or activities particularly relevant to an individual animal are scored by the owner over time.

The results from the readability evaluation, as well as the fact that none of the participants piloting the questionnaire found it difficult to understand or use, support the notion that the questionnaire is feasible for use by almost all horse owners age 12 years and older, equivalent to a U.S. 6th or 7th grade education, and should also be accessible to non-native speakers of English with a linguistic level approximately equivalent to B1 (Intermediate) of the Common European Framework of Reference for Languages [41]. However, for use in clinical and research settings in non-English speaking countries, translated versions would need to be linguistically validated, as has been done for the CBPI [42].

The EBPI was well received by the expert reviews, with many of the comments being directed at making the instrument more specific to OA. This served as a reminder of the need to emphasize that the purpose of the questionnaire is to monitor the degree of chronic pain and the extent to which it is interfering with quality of life, and not to diagnose OA. Several comments suggested defining what scores (thresholds and/or relative increase compared to the last observation on the same horse) would signal a need for veterinary treatment; this will be incorporated into the final instructions for the tool after full clinical validation.

Cronbach’s alpha “reports the extent to which the test score depends upon general and group, rather than item specific, factors” [43]. It is used as a measure of test reliability, but in the sense of the internal consistency of the test items (i.e., how the items within a test relate to each other and contribute to the total score) and not necessarily in the classic sense of consistency of results across time and participants [44]. This makes Cronbach’s alpha an important tool in item selection because it can flag items that may not be contributing to the desired measurement, as well as any redundant items.

While high Cronbach alpha values are desirable, the extremely high value (0.957) obtained in this study is potentially problematic in that it may signal not only good item consistency, but also item redundancy. While such redundancy can be considered a defect if it makes an instrument inefficient [44], inefficiency is not a problem in a questionnaire designed (and indeed shown) to take less than 5 min to complete. The high value does suggest that some items could be eliminated and the instrument shortened. However, given that nearly all the pilot participants suggested adding items to the questionnaire, and only one person identified an item that might be removed, eliminating items could result in reduced face validity, i.e., it might cause horse owners to think that too few questions were being asked to adequately monitor their animal’s condition. In addition, not all of the movement items (e.g., walking on different types of ground surface, going downhill, laying down and getting up) were observed by all owners, necessitating the addition of “Not Observed” to the 0–10 scale. “Not observed” reflected owner language from the focus group discussions.

The interim analysis of test–retest for scoring reliability and validity assessment show that horses with diagnosed OA received high scores on the movement items (Items 5–10) that were consistent with physical restrictions known to be present in arthritic horses. Scores for movement items tended to be higher (often in the 7–10 point range) than scores for posture and facial expression, which were often less than 6 points and frequently in the 0–3 point range. Because the horses in this study have been diagnosed by a veterinarian and the majority are receiving medical treatment, this difference in scores may indicate that chronic OA pain is being successfully managed despite the restrictions in range of motion, leading to a better quality of life for affected animals.

However, the lower posture and facial expression scores may also support previous studies showing owner difficulties in recognizing pain in their horses [26,27,33,34,35]. In addition, a recent study [45] found that humans, whether known to the animal or not, even approaching hospitalized horses reduced the animal’s expression of discomfort behaviors and caused the animal to “perk up”. We have tried to counter such interference by instructing owners to evaluate expression before interacting with their horse and to consider facial expression and posture over the course of a day rather than when they first see their horse (or their horse first sees them). During interviews with owners following return of the questionnaires, two people showed awareness of this problem and said that they would ask friends at the stable to also note their horse’s expression to obtain a more objective evaluation.

Overall, owners/caregivers completing the questionnaire in the currently ongoing validation cohort have found it easy to use, and some have noted that it has improved their ability to recognize signs of pain as well as to be more aware of their horse’s body language. While the purpose of the owner interviews after questionnaire completion is to find out how the OA diagnosis was made and is being treated, along with general information on how the horse is managed and the owner’s discipline and professional or amateur status, interestingly, we are also seeing possible patterns in amateur and professional approaches to OA treatment (i.e., nutraceutical vs. pharmaceutical products) emerge.

An unanticipated finding from our research has been the discovery of distinct owner attitudes toward OA in horses. This largely breaks down into two different views/approaches that owners seem to take. The first, and not entirely surprising owner assessment, is that older horses all have OA to some degree, so there is little to no point in having a veterinarian come out to make a diagnosis. This may result in many older horses not receiving pain treatment that might, in fact, make them more comfortable. The second approach, which we were not expecting, is typified by the comment heard from several stable owners that, “I take good care of my horses, none of them have arthritis”. The thought among some horse owners appears to be that OA is a disease caused by improper management or riding practices and that admitting that a horse has the disease somehow reflects badly on the owner, rider, or caregiver. Again, this likely results in horses not receiving pain management from which they would benefit and also points to a need for an educational campaign to help owners understand that OA is a multifactorial disease that, in general, is not (or not solely) induced by poor riding or (over)training.

Both the differing approaches to treatment and the attitudes toward OA merit further study, which we hope to carry out once the EBPI has been validated.

The main study limitations have to do with participant recruitment. The focus groups used for item creation were smaller than those used for the CBPI. Accordingly, the focus groups were supplemented with key informant interviews. The total number of participants in both types of interviews was slightly over 20 people, which is within the range suggested by Steiner and Norman [28]. Having the participants broken into smaller groups may have been an advantage in that a known limitation of focus group interviews is that “each group really represents a single observation” [46]. This suggests that breaking a limited number of participants into smaller groups may generate more item possibilities, while still allowing for group dynamics and synergy, than combining the participants into one larger group. The fact that no new items appropriate for questionnaire inclusion emerged during the piloting interviews suggests that the focus group and key informant interviews did indeed achieve sampling to redundancy.

Limited participant numbers also raise the issue of the representativeness of both focus group and pilot interview participants. While not all equine disciplines were present among the participants (racing and polo were not represented, for example), essentially no difference in evaluation of questionnaire ease of use and monitoring utility was seen between various equestrian disciplines or between owners of horses in work and those with retired horses. There was, however, a notable difference in item generation and suggested items for inclusion between amateurs and professionals. Amateurs tended to bring up behavioral items such as a horse no longer wanting to do previously “enjoyable” activities, while professionals suggested very specific lateral movements that might indicate a problem. This suggests that either amateur owners tend to pay more attention to the emotional aspect of their horse’s putative pain experience, or professional riders and trainers have a better grasp of physical impairment in their horses. On the other hand, three of the professionals were in fact unaware of the Horse Grimace Scale [21] and the importance of ear position in monitoring pain, suggesting that regardless of professional involvement with horses, there is room for improvement in owner education concerning equine pain expression.

In participant recruitment for the final clinical validation step, we are finding that people are initially very enthusiastic about the project, but then postpone returning the questionnaire or find completing the questionnaire the second time within the appropriate time interval to be difficult. Our difficulties in obtaining completed forms are consistent with the experiences of other organizations currently carrying out surveys. For example, the New York Times estimates that in their polling, only 0.4% of dials currently result in a completed interview, compared with 1.6% in 2018 [47]. The Modern Language Association has seen a 10% drop in university responses to their language enrollment census, with an estimate that without an additional extensive follow-up period that has postponed publication of the survey, the response rate would have dropped by 65–75% [48]. Our impression is that our difficulties in receiving completed questionnaires is the result of a general form of ‘survey fatigue’, since nowadays people are being asked to return surveys after almost every experience, as well as by a fracturing of social bonds and spiraling of work pressure across many sectors caused by the COVID pandemic. We think it is important that other researchers be made aware of possible problems and delays in completing questionnaire- or survey-based research in this post-pandemic era as it has implications for projects aiming at stakeholder engagement, outreach, and education.

## 5. Conclusions

We report the formal development and preliminary assessment of an Equine Brief Pain Inventory for use by owners to assess chronic pain in horses caused by osteoarthritis. The pain monitoring instrument can be completed within 5 min and proved easy to use and interpret by owners with an educational level equivalent to that of 11–12 year olds (U.S. grade 6–7). In the pilot group of owners, the tool was deemed valuable for detection of pain expressions that otherwise might have gone unnoticed. The tool is currently undergoing validation in a larger group of OA-affected and sound control horses. Despite the extended process of completing each step in the established guidelines for validating subjective health measurement scales, we feel it is important to follow the same rigorous method as was used to establish validity and reliability in the human BPI to provide the equine community with a sound instrument that has the potential to be used in practice as well as in future research on equine OA pain relief.

## Figures and Tables

**Table 1:** Range and median scores for horses diagnosed with osteoarthritis (23 horses, age 12–27 years, mean 18.9 years). Because of non-observed items, there were not 23 responses to all items. The summary items (Items 13–15) are not included in the table because these are based on the subjective overall impression of the owner/caregiver.

Item Type/No.	Administration 1		Administration 2	
	Median	Range	Median	Range
Posture				
1	3	0–9	3	0–9
2	3	0–8	3	0–8
Facial expression				
3	2	0–5	2	0–5
4	2	0–7	3	0–7
Movement				
5	7	2–10	7	2–10
6	5	0–10	5	0–10
7	7	0–10	7	0–10
8	7	0–10	7	0–10
9	7	0–10	7	0–10
10	2	0–10	3	0–10
Behavior				
11	3	0–8	3	0–8
12	1	0–7	1	0–7

**Table 2:** Range and median scores for sound horses (5 horses, age 4–16 years, mean 9.8 years). Items 8 and 9 were marked “Not observed” for one horse. The summary items (Items 13–15) are not included in the table because these are based on the subjective overall impression of the owner/caregiver.

Item Type/No.	Administration 1		Administration 2	
	Median	Range	Median	Range
Posture				
1	0	0–3	0	0–3
2	0	0–3	0	0–3
Facial expression				
3	2	0–5	1	0–5
4	1	0–5	1	0–4
Movement				
5	2	0–4	2	0–4
6	0	0–2	1	0–2
7	3	0–3	3	0–3
8	2.5	0–5	2.5	0–5
9	2.5	0–3	1.5	0–3
10	1	0–2	1	0–2
Behavior				
11	2	0–3	2	0–3
12	0	0–5	0	0–4

## Data Availability

The data presented in this study are available in Table 1 and Table 2, Figure 1, and Supplementary Files S1 and S2.

## Supplementary Materials

The following supporting information can be downloaded at: https://www.mdpi.com/article/10.3390/ani14020181/s1, Supplementary File S1: Example Script for Focus Group Discussions; Supplementary File S2: Reviewer Comments and Resulting Revisions.
