# Supplementary material for: Development and Preliminary Validation of an Equine Brief Pain Inventory for Owner Assessment of Chronic Pain Due to Osteoarthritis in Horses
Source: Animals (Basel). 2024 Jan 5;14(2):181. doi: 10.3390/ani14020181 (PMC10812429; doi:10.3390/ani14020181)
Supplement: Supplementary file 1 [file animals-14-00181-s001.zip › Supplementary file S1 Example Script for Focus Group Discussions.pdf]

Supplementary File S1: Example Script for Focus Group Discussions

Please note that the purpose of a focus group is to discover participants' ideas on a particular topic. Therefore, a given session may not follow the example script exactly, because of responses brought up by the participants that might trigger other avenues for discussions. However, the same opening and closing paragraphs will be used.

Start of script: The purpose of this focus group is to discuss how horse owners identify signs of chronic osteoarthritis in their horses and what changes in their animals prompt them to call a vet. The session is being recorded and a transcription will be made of the recording. The recording will be deleted after the transcription to protect your privacy.

Identification of chronic pain in prey animals like horses is extremely difficult because the horse doesn't want you to know about it. OA, in particular, is very difficult to detect in its early stages. So, no one should feel like they've missed something they "should" have seen. The goal is to work together to make identifying pain markers easier and more reliable for everyone and to help our horses.

Let's start with the question, what would make you think that a horse had osteoarthritis?

What behavior or change in behavior would make you call a vet?

Did a specific behavior cause you to call the vet?

How would you monitor the level of pain a horse diagnosed with OA was experiencing?

Breaking things down into smaller parts, do you think there is an analgesic stance specific to chronic OA? An "analgesic stance" a way of standing to relieve pain. The best-known analgesic stance in horses is the rocked back position of a horse with laminitis.

How would the horse hold its neck?

What would the ears look like?

The eyes?

The mouth?

Would there be tension in specific muscles?

What is the difference in your horse's facial expressions on good and bad days?

Do you think a horse with chronic pain would become more withdrawn? More aggressive?

What changes might you see changes during grooming?

How would interactions with people and other horses change?

Would there be changes in the horse's time budget during the day? Would owners be able to notice these?

Are there activities that your horse can no longer do?

What changes might you see in where the horse stands in his/her stall or pasture?

Do you have suggestions about how to monitor how much time a horse spends lying down? Difficulty in lying down and getting back up?

Are there any other behavioral changes you can think of?

What about movement patterns. What differences might you see?

What are some ways of monitoring horse health?

Would you find rating your horse's general activity and enjoyment of life a useful means of monitoring pain?

What words would you use to describe your horses when he/she is feeling bad? Feeling better?

Are there specific activities you would rate to determine quality of life?

We talked about expressions, positioning of body parts, and stance. Do you think scoring these would help determine quality of life?

Are horse owners reluctant to talk about pain?

A Brief Pain Inventory has been created for dogs. It involves highest, lowest, and average pain ratings for the past 7 days, followed by questions about specific activities: lying down and standing/ walking, running, and climbing stairs. Based on our discussion, do you think this would be possible for horses? What behaviors would you include?

Thank you for your participation. If you have any questions or concerns about this discussion, please email me. I would also appreciate hearing your thoughts if you have any additional ideas about equine behaviors linked to chronic OA. Thank you again and I'm turning off the recording now.
