# Supplementary material for: Development and Preliminary Validation of an Equine Brief Pain Inventory for Owner Assessment of Chronic Pain Due to Osteoarthritis in Horses
Source: Animals (Basel). 2024 Jan 5;14(2):181. doi: 10.3390/ani14020181 (PMC10812429; doi:10.3390/ani14020181)
Supplement: Supplementary file 1 [file Supplementary File S2 Reviewer Comments and Resulting Revisions.pdf › Supplementary File S2 Reviewer Comments and Resulting Revisions.pdf]

Supplementary File S2: Reviewer Comments and Resulting Revisions

Item No.	Original Wording	Revised Wording	Reviewer Comments
1	My horse usually **stands**… in a **relaxed manner** and rests its hind legs alternately/as though **reluctant to move**.	My horse usually **stands**… in a **relaxed manner** and may rest its hind legs alternately/in **one position** with **very little movement**.	One reviewer noted that "usually" needed to be defined, and another wrote that "reluctant to move" could be interpreted as not moving forward when lured by a carrot or asked to move from a rope cue. Another observation was that a horse might not rest either hind leg.<br>Other comments were that posture might change with whether the horse was in a stall or at pasture. From the focus group comments and author observations, essentially the same stance is seen in a box and outside.<br>In a box, horses at the higher end of the scale tend to stand at the back of the stall facing the wall, while outside they position themselves away from other animals.<br>Another comment was that it would be surprising if many owners reported a 10 for this item.<br>One reviewer noted that a 'rigid stance' would be unusual for OA and more typical of laminitis or exertional rhabdomyolysis. She also observed that some of the stances cited in the focus groups were more typical of other forms of musculoskeletal pain.<br>Another comment was that the phrase, "rests legs alternately" might cause confusion for owners of horses that weight shift frequently due to pain.
2	When not eating, my horse generally **holds its head and neck… higher than the withers** and is attentive to the surroundings / **at withers level or lower**, not responsive to the surroundings.	When not eating and awake, my horse generally **holds its head and neck… higher than the withers** and is attentive to the surroundings / **at withers level or lower**, not responsive to the surroundings.	Two reviewers noted the need to specify that the horse was awake.<br>That monitoring sleep patterns would be helpful was noted, many owners do not see their horses at night and may never see them lie down. That was discussed in the focus groups.<br>It was also observed that this posture is not specific to OA.<br>Another reviewer noted that this item was asking two questions because of the possibility that a head posture might be normal, but the animal not be responsive to surroundings or that the head carriage was abnormal but the horse responsive to surroundings.
3	My horse's **ears** are… **responsive** to the surroundings/held **back** or **stiffly to the side.**	No change	One reviewer observed that, "horses don't tend to hold their ears back for prolonged periods," and suggested that a specific observation period be given.<br>Three reviewers rated this as an important item, with one saying that this ear position is "one of the most correlated findings with pain."
4	My horse's **eyes** are… **relaxed** and round or oval/**triangular** or **pulled up at the top corner** with wrinkles above the eye.	My horse's **eyes** are… **relaxed** and round or oval/**tense** with a **glazed/zoned out expression**.	Several reviews noted problems with the description, "triangular eyes," and one also said that people have trouble judging eye shape and wrinkles. She suggested using "glazed/zoned out expression instead."<br>One reviewer suggested using a definition based on how open the eye is.<br>Another expert noted that "triangular or pulled up at the top corner with wrinkles about the eye" performs less well when using pain scales.
5	**My horse… moves forward with a loose stride**/is **short striding.**	**My horse… moves forward** with a **loose stride**/is **short striding** with the back or front legs**.**	There were questions about movement when—ridden, walked in hand, free in the field, etc. Because owners may be observing their horses in only one setting, "movement" is being used to cover all the possibilities, so it is deliberately vague. One reviewer noted that having the N/O items was "very important" because of the different ways in which horses are kept.<br>One reviewer asked whether short striding with front or back legs should be specified and also about when

			this observation would be made (e.g., first steps out of the box, coming out of the field, after working for<br>30 minutes, etc.).
6	My horse… stands to have its feet cleaned **as always** with good balance and range of motion/**has changed how** it holds its legs during hoof cleaning, e.g., with poor balance or range of motion.	No change	A reviewer wondered whether owners would understand and/or interpret "range of motion."<br>Another noted that this is not specific to OA.<br>There was a suggestion to add aggressive behavior during hoof cleaning.<br>It was noted that horses with hock OA might lift the leg high when struggling to lower the leg but that could still be classified as reduced ROM. One focus group participant noted that on bad days, her horse with hock OA lifted his leg quite high during hoof cleaning.
7	My horse… **moves easily** into a higher gait/**is reluctant** to move into trot or canter.	No change	One reviewer deemed this item to be "really difficult to interpret" and also noted that transition to lower gaits is equally difficult in terms of joint pain. It was further observed that horses that no longer trot or canter in the pasture have reached the point of advanced OA. Another person noted that this is not specific to OA.<br>A reviewer experienced in questionnaire design suggested that providing some context would make this item more repeatable for owners, so that they were assessing the same movement in the same circumstances in order to detect change.
8	My horse… will walk on **any type of ground**/prefers **soft ground** when available.	No change	Horses with sensitive soles could also receive high scores on this item, but this was rated as an "important question" by one reviewer. Another review also noted that this is more common with foot pain.<br>A further comment was that while less common, a small number of OA horses will struggle with very soft ground (one focus group participant said that her OA horse preferred hard ground).
9	My horse… walks uphill and downhill **with equal ease**/finds walking **downhill** difficult.	My horse walks downhill **easily**/finds walking **downhill** difficult.	"Difficulty going downhill" was a consistent observation from focus group participants, but was not something that reviewers observed. Another reviewer noted that this is common with foot pain. During an expert consultation, participants noted that uphill and downhill is actually two questions and suggested asking about downhill only.
10	New item	My horse can **easily lie down**/**has trouble** lying down and getting up.	Item added at reviewer suggestion.
11	My horse… is **enthusiastic** about activities/no longer **enjoys** or **is not able to do** things it used to do.	My horse… appears to be **enthusiastic** about normal daily activities/no longer **enjoys** or **is not able to do** things he/she used to do.	It was noted by several reviewers that "enthusiastic" is a very subjective description, that there could be other reasons for a horse not to be willing.<br>"Normal daily activities" was added in response to a reviewer comment that this would provide useful context. "It" was changed to "he/she".
12	My horse's **attitude** toward people and other horses… is **unchanged**/has become **withdrawn** or **aggressive**.	My horse's **attitude** toward people and other horses… is **unchanged**/has become **withdrawn**.	It was noted that aggression toward humans is unusual.<br>Another reviewer remarked that this was two questions. As a result, "aggression was dropped from the item.
13	I think my horse's **current level of pain** is … **no pain/severe pain**.	No change	There were several comments about what numbers signal the need for veterinary treatment.
14	I have seen **changes** in the past 7 days… for the **better**/**no change**/for the **worse**.	I have seen **changes** in the scores during the past 7 days… for	The need to specify "changes in what" was noted.

		the **better**/**no change**/for the **worse**.	
15	I would rate my horse's **current quality of life** as… **excellent/restricted/very poor**.	The position (formerly 13, now the final item) and scoring method were changed.	An addition comment was that quality of life can remain good or acceptable with some level of OA pain and also that a horse could have poor quality of life for reasons other than pain. This item was moved to the end so that it could act as an overall impression item. The numerical scale was replaced with Poor, Fair, Good, Very Good, Excellent to mirror the Canine Brief Pain Inventory.
